# Caspar Controls Resistance to *Plasmodium falciparum* in Diverse Anopheline Species

**DOI:** 10.1371/journal.ppat.1000335

**Published:** 2009-03-13

**Authors:** Lindsey S. Garver, Yuemei Dong, George Dimopoulos

**Affiliations:** W. Harry Feinstone Department of Molecular Microbiology and Immunology, Johns Hopkins Bloomberg School of Public Health, Baltimore, Maryland, United States of America; National Institute of Allergy and Infectious Diseases, United States of America

## Abstract

Immune responses mounted by the malaria vector *Anopheles gambiae* are largely regulated by the Toll and Imd (immune deficiency) pathways via the NF-kappaB transcription factors Rel1 and Rel2, which are controlled by the negative regulators Cactus and Caspar, respectively. Rel1- and Rel2-dependent transcription in *A. gambiae* has been shown to be particularly critical to the mosquito's ability to manage infection with the rodent malaria parasite *Plasmodium berghei*. Using RNA interference to deplete the negative regulators of these pathways, we found that Rel2 controls resistance of *A. gambiae* to the human malaria parasite *Plasmodium falciparum*, whereas Rel 1 activation reduced infection levels. The universal relevance of this defense system across *Anopheles* species was established by showing that *caspar* silencing also prevents the development of *P. falciparum* in the major malaria vectors of Asia and South America, *A. stephensi* and *A. albimanus*, respectively. Parallel studies suggest that while Imd pathway activation is most effective against *P. falciparum*, the Toll pathway is most efficient against *P. berghei*, highlighting a significant discrepancy between the human pathogen and its rodent model. High throughput gene expression analyses identified a plethora of genes regulated by the activation of the two Rel factors and revealed that the Toll pathway played a more diverse role in mosquito biology than the Imd pathway, which was more immunity-specific. Further analyses of key anti-*Plasmodium* factors suggest they may be responsible for the Imd pathway–mediated resistance phenotype. Additionally, we found that the fitness cost caused by Rel2 activation through *caspar* gene silencing was undetectable in sugar-fed, blood-fed, and *P. falciparum*-infected female *A. gambiae*, while activation of the Toll pathway's Rel1 had a major impact. This study describes for the first time a single gene that influences an immune mechanism that is able to abort development of *P. falciparum* in *Anopheline* species. Further, this study addresses aspects of the molecular, evolutionary, and physiological consequences of the observed phenotype. These findings have implications for malaria control since broad-spectrum immune activation in diverse anopheline species offers a viable and strategic approach to develop novel malaria control methods worldwide.

## Author Summary

The relationship between malaria parasites and the mosquitoes that transmit them to humans comprises complex molecular interactions including mosquito immune responses. *Anopheles* can mount potent anti-*Plasmodium* immune responses; we show here that the gene *caspar*, which encodes a negative regulator of the immune signaling pathway Imd, controls mosquito resistance to the human malaria parasite. Silencing of this Imd pathway regulator results in complete resistance to human *Plasmodium* in three divergent Anopheline malaria vector species, yet does not cause complete resistance to a rodent *Plasmodium* species, indicating conservation of defense function among mosquito but not diverse parasite species. We also identify a panel of genes that are transcriptionally regulated by silencing of the *caspar* gene. Some of these genes contribute directly to parasite resistance. Finally, we show that the transient immune activation that renders mosquitoes resistant to the human malaria parasite has little to no effect on mosquito fitness as a measure of survival or fecundity under laboratory conditions. In sum, this study shows that the mosquito's immune pathway, Imd, can regulate resistance to *Plasmodium* through immune responses that entail several known anti-*Plasmodium* genes.

## Introduction

The innate immune system of the African malaria vector, *Anopheles gambiae*, is the mosquito's main line of defense against *Plasmodium* parasites and is engaged at multiple stages of parasite infection [1],[2]. As part of this defense, two major signaling pathways, Toll and Imd, transduce the pathogen recognition signal to activate nuclear translocation of the NF-kappaB Rel family transcription factors, which transcriptionally induce effector genes such as antimicrobial peptides [3]. Previous studies have implicated both pathways in the modulation of the mosquito's susceptibility to the rodent malaria parasite *P. berghei*, while a variety of immune effector genes have been shown to mediate the killing of the human pathogen *P. falciparum* [4]–[6]. Interestingly, defense responses to these two *Plasmodium* species have been shown to be quite different, as exemplified by the identification of several parasite species-specific anti-*Plasmodium* genes [6],[7].

In *A. gambiae*, Rel1 (previously Gambif1) is controlled by the Toll pathway and is an analog of the *D. melanogaster* Dif, while Rel2 is controlled by the Imd pathway and is the ortholog of the *D. melanogaster* Relish [4], [8]–[11]. In both species, the negative regulator of the Toll pathway, Cactus, is bound to Dif/Rel1 under naïve conditions, sequestering the transcription factor in the cytoplasm [3],[12]. When Toll is activated, the signal is transmitted through adaptor proteins triggering phosphorylation of Cactus which tags it for degradation. Destruction of Cactus frees Dif/Rel1, which can then enter the nucleus and initiate transcription [13].

The activity of the Imd Rel factor (Relish/Rel2) is held in check by its own inhibitory domains (ankyrin repeats) which can be cleaved by Dredd, a caspase-8 homolog, to cause activation [14],[15]. Regulation of Relish has been shown by Kim and colleagues to be negatively controlled by Caspar, a homolog of the mammalian Fas-associating factor [16]. After observing an increased resistance to Gram negative bacterial exposure and constitutive production of the antimicrobial peptide Diptericin, they determined that Caspar is a negative regulator specific to the Imd pathway that inhibits nuclear localization of Relish by blocking Dredd from cleaving Relish under naïve conditions [16]. Since this is the first and, so far, only study of Caspar, our knowledge of the exact nature and extent of Caspar's inhibitory role is limited.

A variety of *A. gambiae* immune-responsive genes have been identified via gene expression profiling in which mosquitoes infected with bacteria, fungi or malaria parasites have been compared to uninfected controls [6], [17]–[20]. Assays comparing bacteria-challenged and naïve Rel1- or Rel2-depleted mosquitoes have identified both Toll and Imd pathway-regulated immune genes [4],[5]. Many of these pathway-regulated genes, such as *TEP1* (thioester-containing protein 1) and *LRIM1*, have been shown to play key roles in parasite elimination [6],[21],[22]. What is unknown about some of these immune pathways and effectors is the role they play during *P. falciparum* infection and whether they work together or independently while exerting their anti-parasitic action. The complex network of pathogen detection, signal transduction and effector action that ultimately leads to parasite destruction is of utmost interest for the development of vector-based malaria control strategies.

Over 40 *Anopheles* mosquito species act as malaria vectors throughout the world, encountering different environments, food sources, microbial exposures and parasite strains, all of which could impose evolutionary pressures on the insect immune system [23],[24]. A pressing question is whether the anti-*Plasmodium* mechanisms are common or unique among the different *Anopheles* species. This is particularly interesting for anti-*Plasmodium* mechanisms based on immune responses because certain elements of an integrated immune system are conserved while others are highly divergent [25],[26]. Here we have for the first time addressed the role of the Toll and Imd immune signaling pathways in mosquito defense against the human malaria parasite *P. falciparum* not only in *A. gambiae* but also in *A. stephensi* and *A. albimanus* to assess the potential universality of these defense mechanisms. Towards this we have also directly compared the implication of these pathways in the defense against different malaria parasite species.

A finely tuned balance between the capacity of the mosquito's immune system to kill malaria parasites and the impact of *Plasmodium* infection on mosquito fitness exist. A negative correlation has been established between fitness and immune induction but only under chronic conditions which is not the case during transient immune pathway activation [27]. To gain a better insight of the fitness cost of a transient activation of a highly potent immune pathway-mediated anti-*Plasmodium* defense on the mosquito's fitness we have looked at how a *cactus*- or *caspar*-silencing-based immune activation affect the ability of mosquitoes to survive and reproduce.

These novel findings have important implications for understanding molecular and evolutionary components of mosquito immunity to the *Plasmodium* parasite and for the development of vector-based immune-mediated malaria control strategies.

## Results

### *A. gambiae* Caspar is a negative regulator of the Imd pathway–controlled Rel2

**Figure 1:**
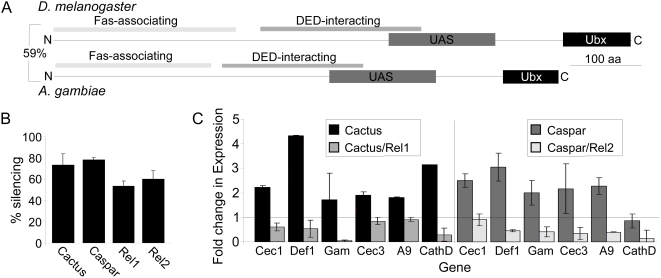
Identification and validation of AgCaspar. (A) Domain comparison between *Drosophila* Caspar (upper diagram) and *Anopheles* Caspar (lower diagram). The horizontal black bar represents the amino acid sequence; black, gray, and white boxes indicate four functional domains of Caspar. Percentage indicates the degree of sequence similarity. DED, death effector domain; UAS, ubiquitin-associated domain; Ubx, ubiquitin-like domain. (B) Silencing efficiency of *caspar*, *cactus*, *rel1*, and *rel2*. Bars represent the percent knockdown compared to controls treated with dsRNA against *GFP* as determined by qRT-PCR. Error bars represent standard deviation of three independent assays. (C) Expression of genes following single or double knockdown in adults. Bars represent fold change in expression compared to *GFP* dsRNA–treated controls. Horizontal gray line represents baseline expression (i.e., a 1∶1 expression ratio between control and silenced samples). Silencing treatment is indicated by the legend. Error bars represent standard deviation across three biological replicates. Cec1, *cecropin1;* Def1, *defensin1;* Gam, *gambicin;* Cec3, *cecropin3;* A9, *ClipA9;* CathD, *cathepsinD*.

The *Anopheles cactus* gene was previously identified through a comparative immunogenomics study of the *Drosophila* and *Anopheles* immune systems and has been functionally confirmed as a negative regulator of Rel1 [3],[5]. Sequence comparison between the putative *A. gambiae caspar* and *D. melanogaster caspar* showed that the mosquito protein sequence shares 43% identity and 59% similarity. Four conserved sequence regions occur similarly in *Anopheles* Caspar as they do in both *Drosophila* Caspar and human FAF1: the Fas-associating region, the DED-interacting region, the ubiquitin-associated domain (UAS) and the ubiquitin-like domain. Fas-associating regions between the two insects' Caspar amino acid sequences were 46% similar, the DED-interacting region, UAS and Ubx domains share 55%, 78% and 81% similarity, respectively (Figure 1A).

To investigate potential functional conservation we combined an RNAi (RNA interference) -mediated gene-silencing approach with expression analyses of putative Imd pathway immune markers. A dsRNA targeting *caspar* or *cactus* was used to silence the respective mRNA transcript in adult female mosquitoes. The average silencing efficiency was 79% for *caspar* and 75% for *cactus*, while *rel1* and *rel2* displayed significantly lower gene-silencing efficiencies of 62% and 55%, respectively, as previously reported (Figure 1B) [5]. The resulting effects on Imd pathway-dependent gene expression were then quantified using quantitative real-time PCR (qRT-PCR) amplification of a panel of antimicrobial peptides (AMPs) previously predicted as Toll or Imd pathway marker genes as well as two genes identified via microarray transcription analyses as regulated by one pathway but not the other [4],[28]. All AMPs showed moderate induction upon Caspar depletion within a 2- to 5-fold range, suggesting that the *Anopheles caspar* is functionally conserved to the *Drosophila caspar* (Figure 1C and Table S1B). To further confirm this relationship and specifically link the Caspar-mediated induction of immune markers to the Rel2 transcription factor, a double gene-silencing assay was performed in which the effect of Caspar depletion was reversed through simultaneous Rel2 depletion. A similar strategy was used for Cactus and Rel1 to ensure consistency. For almost all markers tested, double knockdown reversed the transcriptional increase observed for single knockdown of either *caspar* or *cactus* (Figure 1C), suggesting that Caspar has an influence on Rel2 that affects transcription initiation of key immune genes. A complete reversal of the effects of *caspar* or *cactus* silencing through *rel1* and *rel2* silencing would not be expected, given the transient and partial efficiency of RNAi-mediated protein depletion.

### Resistance of mosquitoes to either the human or rodent *Plasmodium* parasites is differentially dependent on the Toll and Imd pathways

**Figure 2:**
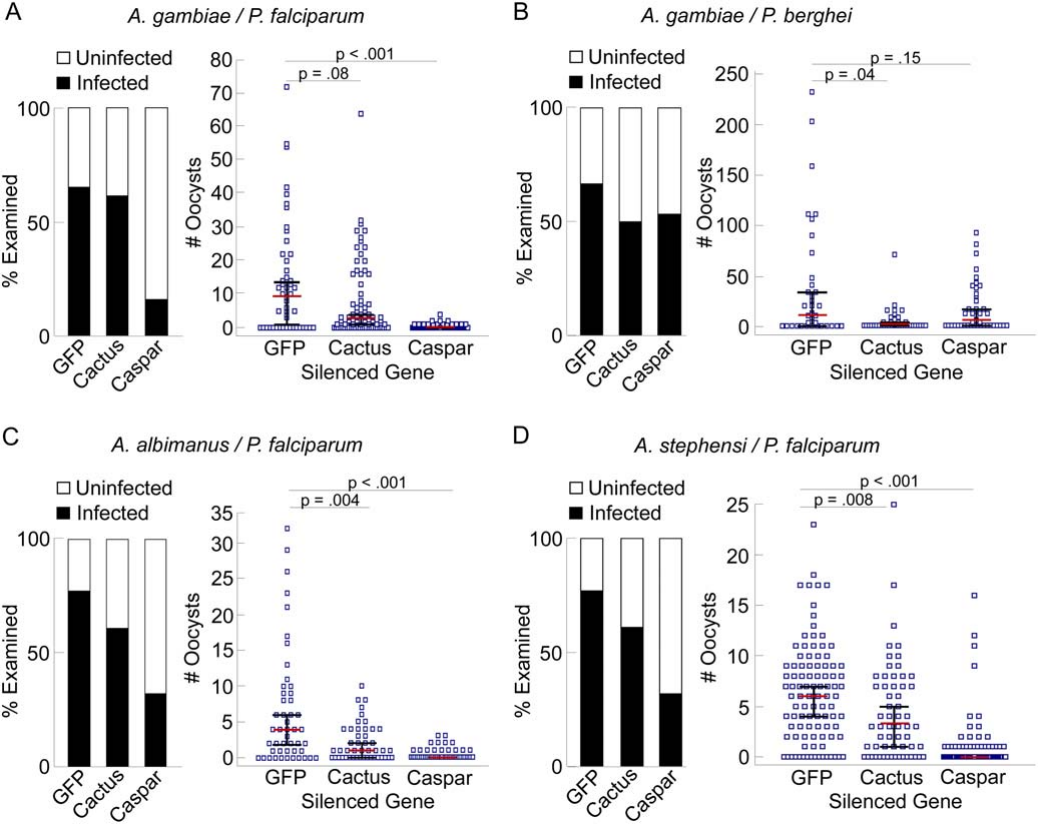
*Plasmodium* infection of Caspar and Cactus-deficient mosquitoes. Prevalence and infection intensity as a measure of *P. falciparum* oocysts in *A. gambiae* (A), *P. berghei* oocysts in *A. gambiae* (B), *P. falciparum* oocysts in *A. albimanus* (C), and in *A. stephensi* (D). For all, the left panel indicates infection prevalence where the bars represent the total population of mosquitoes examined. The filled portion of the bars indicates the proportion of mosquito midguts that stained positive for at least one oocyst; the open portion of the bar indicates the proportion of midguts that were uninfected. The right panel indicates intensity of infection where blue squares represent individual midgut oocyst counts in mosquitoes receiving RNAi treatment against the given genes; the red bars represent the median number of oocysts per midgut, and the black bars indicate the 95% confidence interval.

To investigate the role of these negative regulators in controlling mosquito permissiveness to either the human malaria parasite *P. falciparum* or the rodent malaria parasite *P. berghei*, we depleted female *A. gambiae* of Cactus and Caspar and then fed them on either *P. falciparum*-infected blood through a membrane or on a *P. berghei* infected mouse. Infection levels were compared between the gene-silenced mosquitoes and the GFP dsRNA-treated controls by counting oocysts in the midgut tissue. Strikingly, of the 94 mosquitoes tested in three independent biological assays, only 15 Caspar-depleted mosquitoes (15.5%) allowed a small number of *P. falciparum* to develop to the oocyst stage, as compared to 61.3% of the Cactus-depleted and 65.4% of the control *GFP* dsRNA-treated mosquitoes (Figure 2A, left panel). While the median number of oocysts per gut was 0 in Caspar-depleted mosquitoes, there were still several individuals with low levels of infection. This is likely to have resulted from incomplete gene silencing in these few individuals, which still displayed an unusually low infection level with a median of 1 oocyst (mean  =  1.25 oocysts) (Figure 2A, right panel). This is in stark contrast to that of *GFP* dsRNA-treated mosquitoes, which had a median infection intensity of 9 *P. falciparum* oocysts per midgut (mean  =  21 oocysts) (Figure 2A, right panel). The Cactus-depleted mosquitoes had a median intensity of infection of 2 oocysts (mean  =  8 oocysts) which was just less than one-third of that detected in the control mosquitoes, suggesting that the Toll pathway-controlled Rel1 also exerts an anti-P. *falciparum* effect but not nearly as potent as that of Rel2 (Figure 2A, right panel).

Remarkably, experiments with the rodent *P. berghei* parasite yielded the reverse phenotype: *caspar* silencing had a degree of impact on oocyst development (over 50% reduction) but it was *cactus* silencing that caused a drastic reduction in infection intensity although prevalence was comparable among all groups (Figure 2B). This is in agreement with the phenotype observed by Frolet and colleagues and contributes to an emerging hypothesis that some anti-*Plasmodium* mechanisms, including those utilizing the immune system, show different rates of success when tested comparatively against both *P. berghei* and *P. falciparum* [6],[7].

### Caspar's effect on *P. falciparum* is conserved among three Anopheles species

Bearing in mind that species specificities exist not just within *Plasmodium* but also within *Anopheles*, we were concerned about the possibility that different mosquito malaria vector species use different molecules and/or mechanisms to contain *Plasmodium* infections. We investigated the conservation of the anti-*P. falciparum* phenotype inflicted by *caspar* silencing by replicating silencing and infection assays in two additional anopheline species. Specifically, we looked at *caspar* and *cactus* silencing in *Anopheles albimanus*, a South American vector, and *Anopheles stephensi*, an Asian vector. These species were chosen for their status as major malaria vectors in separate geographical areas and their relatedness to *A. gambiae*. *A. stephensi* belongs to the same sub-genus as *A. gambiae* (*Cellia*) yet distinctly branches from the *A. gambiae* species complex while *A. albimanus* belongs to a different sub-genus (*Nyssorhynchus*) and is distinctly separated from *A. gambiae* in phylogenic trees generated using single copy, ribosomal and mitochondrial sequences as well as those based on morphological characteristics [29]–[31]. Comparisons of these three species provided insight on the conservation of anti-*Plasmodium* defenses between mosquitoes that are separated both spatially and evolutionarily.

Degenerate primers were used to identify species-specific *caspar* and *cactus* sequences for generation of dsRNA. Using these specific dsRNAs and the same gene-silencing and *P. falciparum* infection protocol used for *A. gambiae*, we again observed that *caspar* silencing causes near-refractoriness. Since the phenotype of these mosquito species mirrored that of *A. gambiae*, we hypothesize that *caspar* silencing activates a conserved mechanism for *P. falciparum* inhibition, consistent with its proposed position in the Imd pathway [16]. *dsGFP*-treated controls for *A. stephensi* and *A. albimanus* exhibited median infection intensities of 6 and 4 oocysts per gut, respectively (means for both  =  6 oocysts), while the *caspar*-silenced mosquitoes from each group had a median of 1 oocyst per gut (means ≤1). The effect of *cactus* silencing seems more pronounced in *A. albimanus*, showing a 75% reduction compared to controls, while *cactus*-silencing in *A. stephensi* results in a 50% reduction in median number of oocysts per gut.

### Molecular analysis suggests that the Imd pathway is highly regulated and influences several anti-Plasmodium effectors

**Figure 3:**
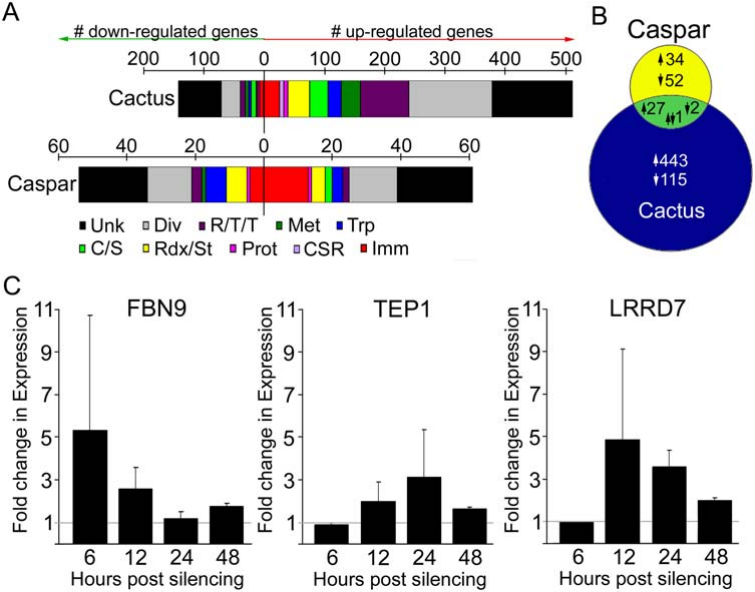
Molecular analysis of *caspar* and *cactus* silencing. (A) Graph indicating functional group distribution of genes regulated by *cactus* and *caspar* silencing. For both upper (Cactus) and lower (Caspar) diagrams, colored sections of graphs correspond to the number of genes either up-regulated (right of zero) or down-regulated (left of zero) for the given functional group (see legend). Unk, unknown; Div, diverse; R/T/T, replication/transcription/translation; Met, metabolism; Trp, transport; C/S, cytoskeletal/structure; Rdx/St, redox/stress; Prot, proteolysis and digestion; CSR, chemosensory; Imm, immunity. Functional groups were predicted using Gene Ontology terms [6]. (B) Venn diagram indicating comparative gene regulation between the two treatment groups. The blue and yellow circles represent the gene expression profiles of the *cactus*- and *caspar*-silenced mosquitoes, respectively, with the overlapping region (green) representing genes that were regulated by both treatments. Numbers with upward-pointing arrows indicate the number of induced genes, numbers with downward-pointing arrows indicate the number of repressed genes, and the single number with one downward-facing and one upward-facing arrow represents the one gene that was enriched after *cactus* silencing and repressed after *caspar* silencing. (C) Temporal expression analysis of anti-*Plasmodium* gene after Caspar depletion. Bars reflect fold change in expression of the indicated gene at 6, 12, 24, and 48 hours after injection of dsRNA against *caspar*. Fold change is determined by real-time quantitative PCR comparison to *GFP* dsRNA–treated controls. Horizontal gray line represents baseline expression (i.e., a 1∶1 expression ratio between control and silenced samples), and error bars indicate standard deviation among three biological replicates. P-values were ≤.05 for: FBN9 at 12 and 48 h.p.i.; TEP1 at 48 h.p.i.; LRRD9 at 6, 24, and 48 h.p.i. Other time points are indicative of trends but did not reach statistical significance among three replicates.

The complexity of an immune response that is more active against one *Plasmodium* species than another and is conserved among *Anopheles* species spurred deeper investigation into the molecular outcome of *caspar* silencing. Identification of molecules affected by *caspar* silencing would provide insight into how the Caspar-driven phenotype is produced. Towards this we investigated the transcriptional influences of *caspar* and *cactus* silencing via a genome-wide comparative transcriptome analysis between Cactus- and Caspar-depleted adult female mosquitoes and control mosquitoes. The robustness of the resulting microarray gene expression data was validated by real-time quantitative PCR (Tables S1A and S1B; Figure S1A and S1B). In total, 588 (472 induced and 116 repressed) genes were significantly regulated upon Cactus depletion and only 116 (61 induced and 55 repressed) were regulated in response to Caspar depletion (Figure 3A and 3B and Tables S3 and S4). In both cases, the majority of the regulated genes belonged to a diverse or unknown functional group; however, there were several remarkable patterns that emerged with regard to other groups of genes (Figure 3A).

The activation of Rel1 via Cactus depletion induced the transcription of 84 genes involved in replication, transcription and translation. The next most-represented functional groups were immunity, redox/stress responses and metabolism. Caspar-depleted mosquitoes also displayed an elevated expression of genes involved in immunity, redox/stress and replication, transcription and translation. Immune genes regulated by either silencing treatment are outlined in Table S2; description of individual genes of interest regulated by these treatments can be found in Text S1. Of note is that several key anti-*Plasmodium* genes such as *TEP1*, *LRRD7* (leucine rich repeat domain protein 7) (also known as *APL2*), and those encoding several FBNs, CLIP domain serine proteases and anti-microbial peptides were regulated by silencing either regulator [6],[21],[32]. Since the microarray depicted only a single time point (3 days post-silencing) and tight temporal regulation of immune genes has been reported [5], we focused more specifically on the transcriptional regulation of genes encoding three key anti-*Plasmodium* factors, FBN9 (fibrinogen immunolectin 9), TEP1 and LRRD7, at 6, 12, 24 and 48 hours post *caspar* dsRNA injection (h.p.i.) to gain a better insight on the temporal nature of this response. Results of these experiments show clearly that these genes are regulated at the transcriptional level by *caspar* silencing and that this regulation is complex and variable among genes. *FBN9* exhibited drastic up-regulation at 6 h.p.i. and its transcripts began to wane as time progresses to reach almost baseline levels (as determined by *dsGFP*-injected controls) at 24 and 48 hours (Figure 3C, left panel). *TEP1* had the opposite expression phenotype where mRNA abundance was still at a baseline level at 6 h.p.i. but climbed incrementally through 24 hours and remained above the baseline level at 48 h.p.i. (Figure 3C, middle panel). *LRRD7* followed a similar pattern as *FBN9* but with a time lag: the spike in *LRRD7* transcript abundance occurred at 12 h.p.i. and began to wane, although expression was still enhanced compared to *dsGFP* controls even at 48 h.p.i. (Figure 3C, right panel).

**Figure 4:**
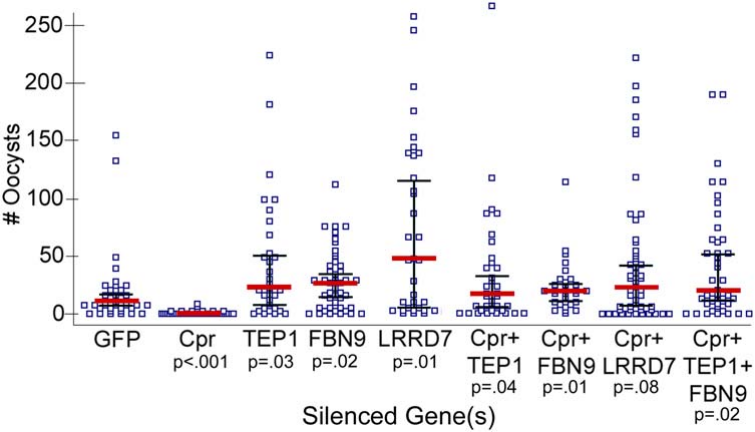
Infection intensities following simultaneous silencing of *caspar* and effector genes. Blue squares represent individual oocyst counts following the indicated RNAi treatment; horizontal red bars represent median number of oocysts per gut, and black bars represent the 95% confidence interval. Assays represent three independent biological replicates and were subject to Mann-Whitney statistical tests. P-values appear below each treatment and refer to that treatment compared to the GFP control. Cpr, Caspar.

To ensure that the transcriptional immune response we observed at the mRNA level was tied to the lack of oocyst development in *caspar*-silenced mosquitoes, we designed assays to test whether TEP1, FBN9 and LRRD7 are indeed part of the *caspar* silencing–mediated anti-*Plasmodium* mechanism. Each of these three genes was silenced independently as well as together with *caspar* in *A. gambiae* females which were then infected with *P. falciparum*. Oocyst counts from the midguts of these mosquitoes are shown in Figure 4. When *TEP1* was silenced alone a median of 42 oocysts would develop while concurrent silencing with *caspar* resulted in 31 oocysts. A median of 27 oocysts developed following *FBN9* silencing alone, 23 oocysts when co-silenced with *caspar* while *LRRD7* silencing permitted a median of 69 oocysts which was muted to 42 with *caspar* silencing. Compared to the *dsGFP*-treated median infection of 18 oocysts and the *caspar*-silenced median of 1, each effector significantly reverses the refractoriness conferred by *caspar* silencing; however, the infections were not as heavy as those that resulted from silencing of any factor alone. We also observe that co-silencing two factors, *FBN9* and *TEP1*, together with *caspar* did slightly increase infection intensity compared to either the *TEP1*+*caspar* or *FBN9*+*caspar* group (median  =  39 oocysts).

### *Caspar* silencing does not impact mosquito fitness

Given the number of genes and variety of functional gene groups that displayed transcriptional induction or repression after Caspar or Cactus depletion (Figure 3), we hypothesized that these *P. falciparum*-resistant mosquitoes might display a reduced level of fitness. Studies of the trade-offs between lifespan and immune defense have shown that mounting an immune response accelerates aging in insects; more specifically, chronic and sustained but not acute and transient Relish (Rel2)-dependent immune signaling reduces the lifespan of *D. melanogaster* [27],[33].

The kinetics of RNAi-mediated gene silencing suggest that pathway induction achieved through silencing of negative regulators would persist for a certain period of time and then diminish over time as the dsRNAs are degraded. Furthermore, our study and that of Frolet et al. suggest that a molecular boosting of basal immunity, prior to infection via pathway activation, causes a different profile of immune gene transcription than persistent infection does [5]. Since the impact of this type of pathway activation on parameters that determine fitness is currently unknown, we asked whether our strategy for immune induction could influence mosquito survival and fecundity under laboratory conditions.

**Figure 5:**
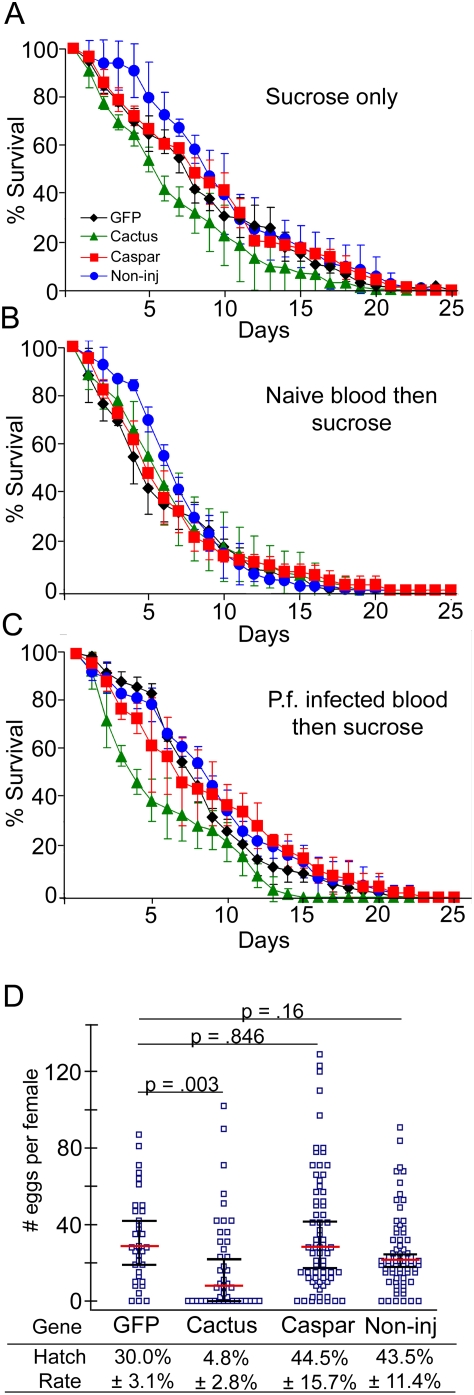
Fitness of *caspar*- and *cactus*-silenced mosquitoes. (A–C) Longevity: Symbols represent the percent of surviving mosquitoes after RNAi-mediated silencing of genes: Caspar (red squares) or Cactus (green triangles); GFP dsRNA treated control (black diamonds), the non-injected control is indicated by the blue circles. The error bars represent the standard deviation for three independent assays. (A) Mosquitoes maintained on 10% sucrose solution; (B) Mosquitoes given a single naïve blood meal via membrane and maintained on 10% sucrose subsequently; (C) Mosquitoes given a single *P. falciparum*–infected blood meal via membrane and maintained on 10% sucrose subsequently. (D) Fecundity: Blue squares represent number of eggs laid by a single female (that received RNAi treatment against the given genes) after a single blood meal; the red horizontal bars represent the median number of eggs laid per female, and black bars represent the 95% confidence interval. Data was subject to Mann-Whitney statistical tests, and plots include data from three independent biological replicates. Hatch rates indicate the average percentage of eggs giving rise to 2^nd^/3^rd^ instar larvae as determined by three biological replicates. Standard deviations derived from these replicates are given as ±. P.f., *P. falciparum*; Non-inj, non-injected.

Both fitness measures were assessed in *A. gambiae* females that had been treated with either dsRNA against *cactus* or *caspar* or control *GFP* dsRNA. Non-injected mosquitoes were also monitored to account for injury-related effects. All groups were tested under three different conditions with regard to nutrition and infection status. Mosquitoes were maintained according to one of three conditions: 1. provision of 10% sucrose throughout the duration of life; 2. given a single naïve blood meal followed by sucrose provision; or 3. given a single *P. falciparum*-laden blood meal followed by sucrose provision. Cactus-depleted mosquitoes given only sucrose showed a slightly impaired survival rate; this was ameliorated by provision of a blood meal but was exacerbated by provision of an infectious blood meal. In stark contrast, Caspar-depleted mosquitoes, regardless of nutritional or infectious status, displayed a similar survival rate to the *GFP* dsRNA-treated controls and non-injected controls. As expected, non-injected mosquitoes tended to fare better than all injected mosquitoes though not significantly (Figure 5A–5C). The number of eggs laid per female and hatch rates of those eggs were also similar between non-injected controls, *GFP* dsRNA-treated controls and mosquitoes treated with *caspar* dsRNA while a quite significant decrease in both measures was observable in the *cactus*-silenced group (Figure 5D).

## Discussion

How the Toll and Imd immune signaling pathways mediate anti-bacterial and anti-*Plasmodium* responses through the Rel1 and Rel2 transcription factors is of key interest, but only recently have some details about pathway regulation become known. Several recent discoveries have shed light on the mechanisms by which pathogens trigger immune gene transcription via Relish/Rel2. Kim and colleagues have shown in *Drosophila* that Caspar is likely to inhibit Dredd-dependent cleavage of Relish [16]. However, in both mosquitoes and mosquito cell lines, Rel2 has been shown to have two active isoforms, one long and one short, with differences in their gene targets [4],[34]. Considering that only the long form, Rel2-F, has inhibitory domains that need to be cleaved for activation, Caspar may only be regulating one branch of Rel2-dependent transcription. Rel2-F has also been shown to be involved in regulating transcription of key Imd pathway marker genes and melanization, and in reducing the number of *P. berghei* parasites in the midgut [4],[28]; however, because our dsRNAs target common domains of both Rel2 forms, we cannot rule out the possibility that Caspar may also be somehow regulating Rel2-S in the mosquito.

The mosquito's immune response against *Plasmodium* is currently under scrutiny as a target for malaria control strategies yet there is much to learn about how immune responses are mounted against *Plasmodium* and what the physiological implications are or how the responses are conserved.

Our data first indicate that manipulation of the mosquito's immune system can produce a near-complete loss of the vector's ability to transmit an unusually virulent strain of the human malaria parasite. Note that we used a *P. falciparum* strain that produces exceptionally high infection levels when compared to naturally acquired *Plasmodium* in the field [35]. This exceptionally virulent strain failed to develop in 85% of the Caspar-depleted mosquitoes, and even those mosquitoes that became infected showed a 94% loss of oocysts when compared to the controls (Figure 2A). Caspar-depleted *A. gambiae* are therefore likely to be completely refractory to natural parasites in the field.

Second, we establish that the reduction in oocysts resulting from depletion of either Cactus or Caspar indicates that both the Toll and Imd immune pathways are involved in the defense against both *P. falciparum* and *P. berghei*, but to different degrees depending on the parasite species (Figure 2A and 2B). This and other studies have shown that activation of the Toll pathway through *cactus* silencing renders mosquitoes almost non-permissive to infection with the rodent malaria-causing parasite *P. berghei* [5], while our data indicate that the same treatment results in an increased but only partial resistance to infection with the human *P. falciparum*. Previous studies have also shown profound differences in the immune responses mounted by *A. gambiae* to *P. berghei* and *P. falciparum* [6],[7]. Our data further support the existence of a distinction between immunity-based resistance to rodent and human malaria parasites and this distinction appears to depend, at least in part, on which Rel factor is activated; i.e. Rel2-based immunity seems most efficient against *P. falciparum* while Rel1-based immunity is most efficient against *P. berghei*. It will be interesting to identify the underlying cause of this distinction; experiments that identify mosquito proteins specifically regulated by either parasite coupled with experiments identifying unique parasite surface molecules may help clarify the distinction.

Third, we observe that the infection-resistant phenotype is not constrained by *Anopheles* species; silencing *A. albimanus* and *A. stephensi caspar* orthologs reproduces the reduction in *P. falciparum* oocysts (Figure 2C and 2D). This, in light of the relative divergence of these mosquito species, brings about important insight into both the evolution of the *Anopheles*-*Plasmodium* interaction as well as the implications for the development of malaria control strategies based on mosquito immunity. Co-evolution is at the crux of the complex interactions between vector, parasite and host. How is it that some interactions can be consistent from one species to another while others are unique? What pressures are exerted by the environment or microbial exposures that precipitate changes or, alternatively, what needs are only met through conservation? Caspar's proposed role in the Imd pathway obviously marks a position in the immune system that is needed by mosquitoes to combat *P. falciparum* and, as a negative regulator of a powerful pathway, may be involved in maintaining the evolutionary balance between controlling the parasite and avoiding chronic immune up-regulation. But how, then, does Caspar depletion circumvent species-specificity? At the cellular and molecular level, the possible differences between species in the *Anopheles* genera are indefinite, meaning slight alterations in any number of molecules, cells or mechanisms could be responsible for variable success of an anti-*Plasmodium* response. We think it is likely that Caspar depletion transcends this limitation because we have targeted a conserved protein in a conserved pathway that leads to the activation of a battery of anti-*Plasmodium* mechanisms. It is certainly possible that some constituents of these downstream mechanisms differ from mosquito species to species but the sum of all parts is a robust overall anti-*Plasmodium falciparum* immune response. This same principle would mean that it is unlikely that *P. falciparum* would acquire resistance to this strategy since there are multiple effectors working in concert. From a malaria control aspect, in this way we can gain refractoriness in a variety of vectors.

Fourth, we have begun to delve deeper into the molecular outcome of *caspar* silencing. Dissection of the downstream battery of immune effectors and how/if they interact is a formidable task and would require extensive experimentation and collaborative efforts. However, using transcriptional and functional genomics, we do establish here that such a mechanism exists and that several key anti-*Plasmodium* genes are involved. The microarray-derived gene expression profiles illustrate that silencing negative regulators activates an arsenal of genes, immune-relevant and otherwise, that may or may not have a direct effect on malaria parasites. Depletion of Cactus resulted in the transcriptional regulation of a more than five-fold higher number of genes than did Caspar depletion, suggesting that Rel1 is a more ubiquitous transcription factor than Rel2 (Figure 3A and 3B). In fact, the Toll pathway has been linked with a variety of processes in development and hemocyte proliferation, and this range of effects was reflected by the broad range of functional gene groups that we found to be influenced by *cactus* silencing (Figure 3A) [12],[36],[37]. Similar observations have also been made in Dif/Dorsal-deficient *D. melanogaster* larvae and other Toll pathway factor mutants that have shown differences in the expression of a large number of non-immune genes, whereas mutations in Imd factors have affected immune gene expression more specifically [37]. Hence, the Toll pathway appears to be a more general and versatile signaling pathway, while Imd is more immunity-specific. Many genes that responded to Cactus depletion are involved in replication, transcription and translation—a likely consequence of the cellular response needed to accommodate the increased activation of a potent transcription factor such as Rel1 and the hemocyte proliferation known to follow Toll pathway activation. Immunity, redox/stress responses and metabolism were also well-represented; immunity and redox reflect Rel1's major role in the response to pathogens and the stress caused by infection while the concurrent enrichment of metabolism- and apoptosis-related genes may provide a transcriptional link between the immune response and recovery from infection.

Although *caspar* silencing influenced transcription of significantly fewer genes, a considerable proportion of regulated genes belonged to the immunity class (see further discussion on individual genes in Text S1). Within this class were genes encoding quite a few known or putative anti-*Plasmodium* effectors that are good candidates for mediating the molecular outcome of *caspar* silencing that results in parasite killing. Some of these candidates, such as FBN9, TEP1 and LRRD7 (APL2), have already been described as performing anti-*Plasmodium* roles and we have shown here that they display a temporally regulated transcription profile when *caspar* is silenced (Figure 3C) [6],[21],[32]. We focused on these 3 genes because of their relevance to the infection phenotype addressed in this study. Using co-silencing techniques, we found that depletion of Caspar cannot confer resistance to *P. falciparum* when FBN9, TEP1 or LRRD7 was co-depleted (Figure 4). These double-silenced mosquitoes harbored similar numbers of oocysts as mosquitoes depleted of the effector alone. Intriguingly, double silenced mosquitoes tended to carry fewer oocysts than the singly silenced which further our theory that a battery of anti-*Plasmodium* genes, and not just one or two effectors, is regulated by Caspar. This was further illustrated by cocktail silencing experiments in which Caspar, along with two or more effectors were depleted. These triple gene-silenced mosquitoes showed a slightly greater (though not additive) infection intensities compared to those with just *caspar* and a single effector silenced, suggesting a concerted effort occurring either downstream or in parallel to Caspar. That the Imd pathway could control regulation of a fibrinogen-domain-containing molecule and a complement-like molecule that together interact to combat invading parasites is not unlikely. In vertebrates, ficolins are the fibrinogen-like domain containing pattern recognition receptors that activate the lectin complement pathway [38],[39]. Evidence of lectin pathway components in chordates and jawless vertebrates suggests that it is the most ancient complement activation pathway [40],[41]. The precedence set in the vertebrate system for these two molecules to cooperate to destroy pathogens as well as the observation that both TEP1 and FBN9 co-localize to the parasite surface justify speculation that a lectin complement pathway–like mechanism may also exist in mosquitoes ([21] and Dimopoulos lab, submitted).

Finally, to investigate the potential fitness impact of this highly potent anti-*Plasmodium* defense, we performed several proof-of-principle assays to determine if longevity or fecundity could be influenced by the RNAi-mediated transient immune response activation. We concede that this is by no means a thorough study of how Imd and Toll activation pathway activation impact fitness, but it provides baseline indications on any obvious physiological consequences of this immune boosting. The fact that *caspar*-silenced, but not *cactus*-silenced, mosquitoes lived as long as controls indicates that the products of the Imd, but not the Toll, pathway have a negligible effect on longevity under these conditions. Since our gene expression data indicated that *cactus* silencing influences five times more genes than does *caspar* silencing, it may be that the Toll pathway's transcriptional output is more costly than that of the more immune-specific Imd pathway. This difference could also reflect a greater toxicity of Toll products in the host. Reduced fecundity could also impart a reduction in fitness yet we observed that the same number of viable offspring were produced per *caspar*-silenced female as *dsGFP*-treated or non-injected controls; taken with the longevity data, we conclude that fitness by these measures and in this environment is unimpaired by the transient Rel2 –mediated immune response. The contrasting fitness cost of *cactus*-silencing suggests that manipulation of the Toll pathway may be too detrimental to mosquito fitness for its use in a malaria control strategy, though it would be quite interesting to continue investigating how Toll pathway activation impacts fitness and how insects negotiate the cost/benefit of Toll pathway activity. On the other hand, that Caspar manipulation does not confer a noticeable fitness cost warrants future evaluation in more realistic contexts and further analysis of its molecular mechanism. We do suspect that fitness costs will be less obvious in this context than in others, since the immune induction here was not sustained for a lifetime but was relatively temporary because of the kinetics of the RNAi process. This situation is in accordance with reports showing that sustained NF-kappaB-dependent chronic immune responses are correlated with a shorter life while temporary acute responses are not [27]. We also questioned whether this analysis could have become compromised by the experimental method that required injection of dsRNA which also could facilitate introduction of bacteria that could alter survivorship; the *caspar* dsRNA treated mosquitoes may in this case experience an advantage due to immune activation We must use this technique as gene silencing and over expression by other methods are prohibitively difficult in *A. gambiae*. As a control, hemolymph from mosquitoes injected with dsRNA against *caspar* and GFP as well as unwounded mosquitoes was perfused with saline and plated on LB agar at sterile conditions. Resulting colony counts indicated that individual mosquitoes from all groups contained a large range of bacterial quantities and no significant differences were found among groups suggesting that if bacteria are introduced into the hemocoel during injection, they are in negligible amounts or are unable to survive and/or proliferate (data not shown).

Upon *caspar* silencing, we did not observe any spontaneous melanization that was the compelling phenotype for the discovery of Caspar deficiency in *Drosophila* [16]. It is likely that the absence of melanization in mosquitoes simply reflects differences in how deficiencies are generated; a P-element insertion in *Drosophila* can cause Caspar loss of function throughout the body for a lifetime while the RNAi-mediated silencing strategy we employed is transient and causes differing levels of transcript degradation in different tissues. Because of this, we can not rule out that a constitutive and thorough Caspar loss of function would cause a similar hyper-melanization phenotype in mosquitoes.

By manipulating Caspar and Cactus in the present study, we were able to produce a transcriptional immune response that potentially involved a variety of anti-*Plasmodium* factors. Thus, *caspar* silencing was probably so effective against *P. falciparum* because it simultaneously enhanced an entire battery of relevant effectors and mechanisms. Global induction is advantageous for development of control strategies, since it avoids reliance on a single gene and the associated limitations in effectiveness: that it may only be effective against a certain parasite stage or strain and that resistance may more easily develop. This may also be why, as a physiological and biochemical mechanism, immune system activation produces a more extensive reduction in parasite burden than synthetic methods. If our strategy of depleting Caspar proves to be an effective way of limiting vector competence, short-term immune induction could be achieved through infection-inducible promoters that drive recombinant Rel2 expression. A further understanding of the impact of immune pathway activation on the mosquito's fitness will necessitate not only the development of an injury-independent mechanism but also the assessment of fitness under field conditions.

## Materials and Methods

### Identification of *A. gambiae* Caspar

Peptide sequences for *Drosophila* Caspar (CG8400) and its predicted *A. gambiae* ortholog were retrieved from Ensembl and subject to alignment using BLAST and Clustal W and the BLOSUM62 scoring matrix. Sequence alignment achieved an expected value of 5e^−150^. Domain similarity was determined by BLAST alignment of amino acid sequences of *Drosophila* Caspar regions as defined by Kim et al. [16] against the entire *Anopheles* Caspar sequence. Domain regions were then verified using the SMART database version 5.1 [42].

### Mosquito rearing

*A. gambiae* Keele strain, *A. albimanus* Santa Tecla and *A. stephensi* mosquitoes were maintained on a 10% sugar solution at 27°C and 80% humidity with a 12-h light/dark cycle according to standard procedures [43]. For all mosquitoes, ∼250 larvae per 30×34 cm tray were reared with daily addition of cat food pellets and a ground fish food supplement upon water change. Adults were reared in a 20×20×20 cm^3^ cage and provided 10% sucrose.

### RNAi gene silencing

Assays were performed according to standard protocol [6]. Sense and antisense RNAs were synthesized from ∼300–600 bp PCR-amplified gene fragments using the T7 Megascript kit (Ambion) and primers indicated in Text S1. About 69 nl dsRNA (2–3 µg/µl) in water was introduced into the thorax of cold-anesthetized 2–4 day old female mosquitoes by a nano-injector (Nanoject, Drummond) with glass capillary needles according to an established methodology [21].

### Real-time quantitative PCR expression analysis

Assays were performed according to standard protocol [6]. Total RNA from adult females was extracted using the RNeasy kit (QIAGEN), quantified using a Beckman DU640 spectrophotometer and subjected to reverse transcription using Superscript III (Invitrogen) with random hexamers. Real-time quantification was performed using the QuantiTect SYBR Green PCR Kit (Qiagen) and ABI Detection System ABI Prism 7000. Primer sequences are given in Text S1. All qPCR reactions were performed in triplicate; to check for the specificity of the PCR reactions, melting curves were analyzed for each data point. The levels of expression in gene-silenced samples were determined by normalizing cDNAs using the ribosomal protein S7 gene and comparing to controls treated with dsRNA against GFP. P-values were determined using a student's T-test.

### Challenge with *Plasmodium*

*P. falciparum* and *P. berghei* infections were administered according to standard protocol [6]. For *P. falciparum* infections, mosquitoes were fed on NK54 gametocytes in human blood through a membrane feeder at 37°C 4 days after dsRNA treatment. Unfed mosquitoes were removed within 24 h after feeding, and the rest were maintained at 24°C for 7 days. For *P. berghei* infections, mosquitoes were fed on Swiss Webster mice infected with the wt Anka 2.34 strain of *P. berghei* at 21°C 4 days after dsRNA treatment. Unfed mosquitoes were removed from the group within 24 h after feeding and the rest were maintained at 21°C for 14 days. For both infections, mosquito midguts were dissected and stained with mercurochrome, and oocyst numbers were recorded using a light-contrast microscope (Olympus). Each assay was done with at least 25 mosquitoes, and data represent the results of three independent assays. P-values were determined using a Mann-Whitney test.

### Sequencing of *A. albimanus* and *A. stephensi* caspar and *cactus*

Using known sequences from *A. gambiae*, *D. melanogaster* and *A. aegypti*, degenerate primer pairs (with sequence bias toward *A. gambiae*) were designed to amplify regions spanning the entire transcript of either gene. Regions were amplified from total cDNA from each mosquito species using standard PCR protocol and cycling program. PCR products were excised from a 1% agarose gel, extracted (Qiagen Qiaquick gel extraction kit), sequenced (Applied Biosystems 3730×l DNA Analyzer), and aligned to *A. gambiae* sequenced using BLAST and Clustal W and the BLOSUM62 scoring matrix. Compared to *A. gambiae*, sequences of regions of *A. albimanus cactus* and *caspar* had identities of 77–85% and 77–100%, respectively while regions of *A. stephensi cactus* and *caspar* had identities of 70–92% and 80–93%, respectively. Regions used for generating double-stranded RNA achieved the following nucleotide identities: *A. albimanus cactus*, 77%; *A. albimanus caspar*, 79%; *A. albimanus cactus* 75% and *A. stephensi caspar*, 92%.

### Microarray analysis

Assays and analysis were performed according to standard protocol [6]. All arrays performed using female *A. gambiae* at 3 days post silencing. Gene expression values were compared between Cactus- and Caspar-depleted adult female mosquitoes and *dsGFP*-treated controls. Total RNA was extracted from 10–15 whole mosquitoes 3 days after dsRNA treatment using the RNeasy kit (QIAGEN). Quantification of RNA was performed using a Beckman DU640 spectrophotometer, and quality was assessed by RNA Nano LabChip analysis on an Agilent Bioanalyzer 2100. Probes were synthesized with 2–3 µg RNA using the Agilent Technologies low-input RNA labeling kit according to the manufacturer's instructions. Hybridizations were done with the Agilent technologies in situ hybridization kit according to manufacturer's instructions. Arrays were washed then dried using pressurized air. Microarray scanning was done with an AXON 4200AL scanner, with the laser power set to 60% and the PMT gain adjusted manually to maximize effective dynamic range and avoid spot saturation. Images were analyzed with Genepix 6.0 software to determine spot size, location and quality, and potentially confounding spots were manually removed from the analysis. The data were then processed with TIGR MIDAS software [44]. The intensity threshold was set to 100 for both Cy5 and Cy3 channels, and the signal-to-background cutoff was set to 2.0 in both channels. Data for each experimental condition were derived from three biological replicates. The median spot intensities for each spot meeting the above criteria were normalized according to a LOWESS normalization method. The TIGR MeV software [44] was then used to Log2 transform the Cy5/Cy3 ratios and perform t-tests with the following parameters: mean value to be tested against  =  0, the Welsh approximation method for degree of freedom calculation, P-values based on t-distribution, the overall alpha (critical p-value) set to 0.05, no p-value correction. According to established methods, a cut-off value for the significance of the gene regulation was set to 0.8 in a log2 scale (1.74-fold ratio) [45]. Values for genes that had a significant p-value for one experimental set were included in other experimental sets, providing that the direction of regulation was consistent and the value was within a range of <0.5 fold.

### Longevity assay

Two-day-old adult female *A. gambiae* were treated with dsRNA against *cactus*, *caspar* or *GFP* as described, then incubated at 27°C with 70% humidity while being maintained on sterilized 10% sucrose solution. Injected mosquitoes and non-injected mosquitoes from the same generation were monitored simultaneously to control for injection-induced mortality. All cohorts were monitored daily for survival, and dead mosquitoes were removed each day. Monitoring continued until all mosquitoes had perished. Percentages represent the mean survival between 3 biological replicates of 50 mosquitoes each. Statistical significance was determined using Kaplan-Meier and log-rank analyses.

### Fecundity assay

For each of three biological replicates, at least 30 two-day-old female mosquitoes were treated with dsRNA then at 3 d.p.i. (days post injection) allowed to feed on human blood through an artificial membrane system for 30 minutes. Fed mosquitoes were transferred to individual wax-lined cardboard cups outfitted with cotton soaked in 10% sucrose solution and an oviposition cup filled with water and lined with filter paper. Individual chambers were incubated under normal rearing conditions. Eggs oviposited on filter paper were counted after 2 days using light microscopy. Those females with no eggs on day 2 were maintained and filter papers were examined on day 3. After each count, eggs were submerged *en masse* in a standard larval pan for rearing according to standard methods (see rearing methods). Second or third instar larvae were counted and removed from larval pan daily. Statistical significance of oviposition was determined using Mann-Whitney and statistical significance of hatch rates was determined using one way ANOVA (analysis of variance).

### Hemolymph perfusion and bacterial colony counts

For each of three biological replicates, at least 10 two-day old female mosquitoes were microinjected with dsRNA or reserved as non-injected controls. Three days post silencing, mosquitoes were surface sterilized with 70% ethnol and PBS and sterile PBS was perfused through the thorax and collected with a sterile needle according to previous established protocol (ref). Collected hemolymph was serially diluted in PBS and dilutions were each plated on LB agar. Plates were sealed and kept in insectary conditions for 2 days then colonies were counted. Number of bacteria per mosquito was determined using an average of all serial dilutions for that individual.

### Ethics Statement

All animals were handled in strict accordance with good animal practice as defined by the relevant national and/or local animal welfare bodies, and all animal work was approved by the appropriate committee.

## Supporting Information

Figure S1 Validation of gene expression analyses of *cactus*- and *caspar*-silenced mosquitoes. (A,B) Validation of microarray using qRT-PCR. Array-derived gene expression values are plotted on the x-axis while averages of three real-time PCR-derived gene expression values from biological replicates are plotted on the y-axis. Each point represents the log-transformed values for one tested gene; which are given in Tables S1A and S1B. Line of best fit (Microsoft Excel) is included. (A) Gene expression following *cactus* silencing (compared to GFP). The best-fit linear-regression analysis (*R^2^*  =  0.770), and the slope of the regression line (*m*  =  1.15) demonstrated a high degree of correlation between gene expression magnitudes determined via array and real-time assays. (B) Gene expression following *caspar* silencing (compared to GFP). The best-fit linear-regression analysis (*R^2^*  =  0.772) and the slope of the regression line (*m*  =  .64) demonstrated a reasonably high degree of correlation between gene expression magnitudes determined via array and real-time assays.
(0.45 MB TIF)

Text S1 Supplementary Text
(0.05 MB DOC)

Table S1 Gene expression in *cactus-*, *caspar-*, *cactus/rel1-* and *caspar/rel2*-silenced mosquitoes.
(0.03 MB PDF)

Table S2 Immune genes regulated by negative regulator silencing.
(0.03 MB PDF)

Table S3 Cactus gene-silenced transcriptome expressed as log2 transformed *cactus* gene-silenced/GFP dsRNA expression ratio.
(0.09 MB XLS)

Table S4 Caspar gene-silenced transcriptome expressed as log2 transformed caspar gene-silenced/GFP dsRNA expression ratio.
(0.03 MB XLS)
