# Supplementary figures and images for: Caspar Controls Resistance to Plasmodium falciparum in Diverse Anopheline Species
Source: PLoS Pathog. 2009 Mar 13;5(3):e1000335. doi: 10.1371/journal.ppat.1000335 (PMC2647737; doi:10.1371/journal.ppat.1000335)

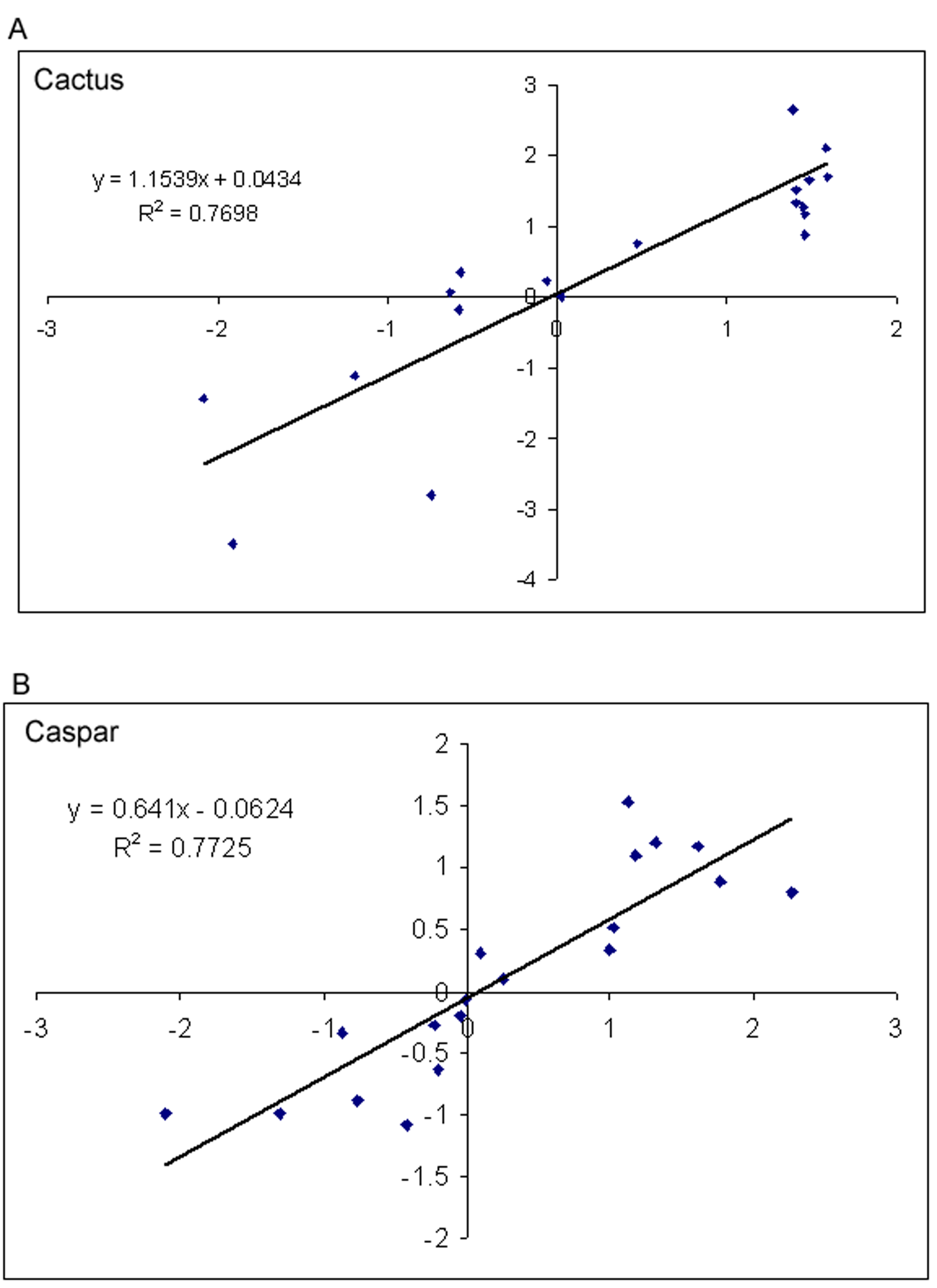

Supplement: Figure S1 — Validation of gene expression analyses of cactus- and caspar-silenced mosquitoes. (A,B) Validation of microarray using qRT-PCR. Array-derived gene expression values are plotted on the x-axis while averages of three real-time PCR-derived gene expression values from biological replicates are plotted on the y-axis. Each point represents the log-transformed values for one tested gene; which are given in Tables S1A and S1B. Line of best fit (Microsoft Excel) is included. (A) Gene expression following cactus silencing (compared to GFP). The best-fit linear-regression analysis (R2 = 0.770), and the slope of the regression line (m = 1.15) demonstrated a high degree of correlation between gene expression magnitudes determined via array and real-time assays. (B) Gene expression following caspar silencing (compared to GFP). The best-fit linear-regression analysis (R2 = 0.772) and the slope of the regression line (m = .64) demonstrated a reasonably high degree of correlation between gene expression magnitudes determined via array and real-time assays. (0.45 MB TIF) [file ppat.1000335.s001.tif]
