# Supplementary material for: Caspar Controls Resistance to Plasmodium falciparum in Diverse Anopheline Species
Source: PLoS Pathog. 2009 Mar 13;5(3):e1000335. doi: 10.1371/journal.ppat.1000335 (PMC2647737; doi:10.1371/journal.ppat.1000335)
Supplement: Text S1 — Supplementary Text (0.05 MB DOC) [file ppat.1000335.s002.doc]

Supplementary Text

**Primers for RNAi assays:**

CactusF: 5’-TAATACGACTCACTATAGTAACACTGCGCTTCATTTGG-3’

CactusR: 5’-TAATACGACTCACTATAGGCCCTTTTCAATGCTGATGT-3’

Caspar F: 5’-TAATACGACTCACTATAGCCGCTTTTCTAAACGCTGTC-3’

CasparR: 5’-TAATACGACTCACTATAGAAACAGGTTGCATGTGTGGA-3’

Rel1F: 5′- TAATACGACTCACTATAGATCAACAGCACGACGATGAG-3′

Rel1R: 5′- TAATACGACTCACTATAGTCGAAAAAGCGCACCTTAAT -3′

Rel2F: 5′- TAATACGACTCACTATAGCGGAGAAGTCGAAGAAAACG-3′

Rel2R: 5′-TAATACGACTCACTATAGCACAGGCACACCTGATTGAG -3′

AnAlbCasparF: 5’: TAATACGACTCACTATAGGCGAAGCGACAGAAGGAG-3’

AnAlbCasparR: 5’: TAATACGACTCACTATAGTCTTAAGTCTCTGCGAGGCCAGCTTGAGAT-3’

AnAlbCactusF: 5’: TAATACGACTCACTATAGCTGAACATTCAGAACGAT-3’

AnAlbCactusR: 5’: TAATACGACTCACTATAGCGGAGATTTTCCTTCCCT-3’

FBN9F: 5′- TAATACGACTCACTATAGCCAAGATGTCGGGCAAGTAT -3′

FBN9R: 5′- TAATACGACTCACTATAGTTGTGGTACGTCAGCGAGTC -3′

TEP1F: 5′- TAATACGACTCACTATAGGTTTGTGGGCCTTAAAGCGCTG -3′

TEP1R: 5′- TAATACGACTCACTATAGGACCACGTAACCGCTCGGTAAG -3′

LRRD7F: 5′- TAATACGACTCACTATAGTCGGTGAGCAACAGTTTGAC -3′

LRRD7R: 5′- TAATACGACTCACTATAGCTTCATTCCCGCTAATGCTC-3′

Underlined sequence corresponds to T7 promoter sequence. AnAlb primers refer to RNAi primers used specifically for *A. albimanus* and *A. stephensi*.

**Primers for qRT-PCR:**

CasparVerifyF: Same as RNAi Forward Primer

CasparVerifyR: 5’-GAACGGCTGCGCTTTAACA-3’

CactusVerifyF: Same as RNAi Forward Primer

Cactus VerifyR: 5’-TCGTTCAAGTTCTGTGCAAGTGT-3’

Cecropin1F: 5’-AGACCAACCAACCACCAAAC-3’

Cecropin1R: 5’-GTTAGCAGAGCCGTCGTCTT-3’

Gambicin1F: 5’-TGCGAGATGTAAAAGCATCG-3’

Gambicin1R: 5’-CCAACGTCTGGCACTGATTA-3’

Defensin1F: 5’-GCGGTTCCAAAGTTCCGACA-3’

Defensin1R: 5’-AGCGGGACACAAAATTGTTC-3’

CLIPA9F: 5’-GTTGGGGACTGGACGTTAGA-3’

CLIPA9R: 5’- GTACCCTTCGATCGTGTCGT-3’

CathepsinD: 5’- TTCTCGGATATGCCGATTTC-3’

CathepsinD: 5’- TCTCGAACGAGGACGACTTT-3’

Verify Primers were used to verify silencing efficiency. Other silencing treatments were validated previously [1, 6].

**Supplementary Figure and Table Legends**

**Table S1**: Gene expression following single or double knock down of negative regulators and Rel factors Column 1: Gene name; Column 2: Array-derived gene expression values in *cactus-* or *caspar*-silenced mosquitoes; Column 3: Real-time PCR-derived expression values in *cactus*- or *caspar-*silenced mosquitoes; Column 4: Real time PCR-derived gene expression values in *cactus/rel1* or *caspar/rel2* double silenced mosquitoes Numbers represent ratio of silenced to control mosquitoes All values represent the average of 3 biological replicas A) *cactus*-silenced groups B) *caspar*-silenced groups

**Table S2**: Microarray-derived gene expression values for immune genes.

Column 1 indicates gene name, Column 2 indicates the Ensembl transcript ID for each gene and Column 3 indicates the log transformed fold change in gene expression due to: A) *cactus* silencing or B) *caspar* silencing

**Table S3**: *cactus* gene silenced transcriptome expressed as log2 transformed *cactus* gene silenced/GFP dsRNA expression ratio.

**Table S4**: *caspar* gene silenced transcriptome expressed as log2 transformed *caspar* gene silenced/GFP dsRNA expression ratio.

**Analysis of individual genes regulated by Cactus and Caspar depletion**

Caspar depletion regulated immune genes with diverse functions, such as those encoding the Clip domain serine protease CLIPB17, the serine protease inhibitor serpin5, pro-phenoloxidase1, several fibrinogen immuno-lectins (FBNs), two scavenger receptors (SCRs) and two peroxidases, as well as several genes involved in apoptosis, were regulated by knockdown of *cactus* but not *caspar*. Genes up-regulated following *caspar* but not *cactus* silencing encode a serine protease (CLIPB4), a putative homolog of mammalian MD-2 (AgMDL8), SCRB1 and an FBN yet down-regulated a serine protease and FBN9. The 27 genes that were up-regulated upon the depletion of either factor included 8 immune genes: 3 AMPs (*Cec1*, *Cec3* and *Def1*), 3 serine proteases (*CLIPA9*, *CLIPD4* and one novel), *TEP3* and *FBN37*. Both treatments repressed two genes, one of which is related to immunity. Only one gene displayed the opposite pattern of regulation; the anti-*Plasmodium* factor *LRRD7* (leucine rich repeat domain 7, also known as *APL2*) showed enrichment upon *cactus* silencing and repression upon *caspar* silencing (Figure 3 and Table S1).

Cactus depletion specifically induced transcription of the genes for CLIPB17 serine protease, which is known to promote parasite melanization and SRPN5, a serine protease inhibitor, yet down-regulates that of phenoloxidase enzyme PPO1 [46]. This signature is likely to represent a regulatory mechanism for melanization-dependent defenses directed by the Toll pathway. Genes encoding three programmed cell death proteins, Bax inhibitor, PCD4 and CathepsinD, were also influenced by *cactus* silencing which may relate to the suggested role of Toll-like receptors as sensors for autophagy in mammals though the signaling for insect autophagy is as yet unknown [47]. Two peroxidases are increased upon *cactus* silencing, one of which is up-regulated in the *A. gambiae* midgut during *P. berghei* infection, possibly as part of the apoptotic response of midgut epithelial cells [48].

*caspar* silencing influenced transcription of significantly fewer immune genes, which is a likely indication that this negative regulator affects a certain branch of the Imd pathway: the Dredd-dependent activation of Rel2 [16, 49]. This selectivity, together with our use of adult mosquitoes (containing a variety of immune and non-immune tissues) instead of cell lines and a timepoint reflecting late expression, were most likely the reasons for the absence of detectable differential expression of some previously reported target genes of the Imd pathway, such as *PGRP-LC* and *LRIM1*, following Caspar depletion. Enhanced expression was, however, observed for the antimicrobial peptide genes *cec1, cec3* and *cef1,* which are targets of the Imd pathway and may exhibit specificity with regard to the Rel isoform to which they respond [4, 28]. We also note that the expression of the anti-microbial peptide *gambicin* (*gam1*) following silencing of either *cactus* or *caspar* did not pass the induction threshold we used for array analysis but was picked up as weakly induced by the sensitivity of real time PCR.

The association of a highly potent anti-*Plasmodium* activity with Caspar depletion suggests activation of Rel2 results in transcriptional activation of anti-*Plasmodium* effectors. A limited number of such effectors that are controlled by the Rel1 and Rel2 factors have already been identified [4-6]. Caspar depletion would therefore be expected to cause transcriptional activation of genes responsible for this dramatically refractory phenotype and, similarly, Cactus depletion would be expected to induce genes mediating its observed infection phenotype. By specifically targeting these molecules that are unique to one Imd branch or the other, we can begin to understand the complexity of the Imd pathway and it’s regulation of anti-*Plasmodium* defense (see main text).

The overlaps between Rel1- and Rel2-induced gene expression profiles were extensive, including several innate immune genes (Figure 3B). Three anti-microbial peptides, *cec1*, *cec3* and *def1* were up-regulated by both Cactus and Caspar RNAi-mediated depletion. This result is not surprising, since dual regulation of anti-microbial peptides by both the Toll and Imd pathways has been shown in *Drosophila* and mosquito cell lines [16, 50, 51]. Four serine proteases, including CLIPA9 and CLIPD4, showed enrichment in response to the activation of both Rel factors. Serine proteases, such as those with Clip domains, are responsible for the signal amplification that occurs between pathogen recognition events and the activation of the Toll or Imd pathways and are also linked to the melanization of parasites and other foreign bodies [46, 52, 53].

Both Cactus- and Caspar-depleted cohorts showed elevated expression of the TEP3 gene, a member of the thioester-containing protein family that also includes TEP1, a potent anti-*Plasmodium* molecule, has been reported to be up-regulated during bacteria and malaria infection [3, 54]. FBN37, a putative pattern recognition receptor, is another immune gene that was enriched in both gene expression profiles. The enrichment of transcripts encoding the *frizzled* protein in both Cactus- and Caspar-depleted mosquitoes is intriguing, since frizzled has been implicated as a key mediator of melanization and the actin polymerization that occurs around the rodent *P. berghei* parasites in the mosquito midgut, and may be linked to TEP1 activity [55]. One anti-Plasmodium factor, LRRD7 (APL2), was induced by *cactus* silencing but repressed by *caspar* silencing (see main text for further analysis). LRRD7 (APL2) is of particular significance because this gene is located in a region conferring resistance to *Plasmodium*, and silencing of this gene causes *P. falciparum* infection to increase by two-fold (see main text for further analysis) [6, 32].

46. Volz, J, Muller, HM, Zdanowicz, A, Kafatos, FC and Osta, MA (2006) A genetic module regulates the melanization response of *Anopheles* to *Plasmodium.* Cell Microbiol 8: 1392-405.

47. Xu, Y, Jagannath, C, Liu, XD, Sharafkhaneh, A, Kolodziejska, KE and Eissa, NT (2007) Toll-like receptor 4 is a sensor for autophagy associated with innate immunity. Immunity 27: 135-144.

48. Kumar, S, Gupta, L, Han, YS and Barillas-Mury, C (2004) Inducible peroxidases mediate nitration of *Anopheles* midget cells undergoing apoptosis in response to *Plasmodium* invasion. J Biol Chem 279: 53475-82.

49. Zou, Z, Evans, JD, Lu, Z, Zhao, P, Williams, M, et al (2007) Comparative genomic analysis of the Tribolium immune system. Genome Biol 8:R177.

50. De Gregorio, E, Spellman, PT, Tzou, P, Rubin, GM and Lemaitre B (2002) The Toll and Imd pathways are the major regulators of the immune response in *Drosophila.* EMBO J 21: 2568-79.

51. Tanji, T, Hu, X, Weber, AN and Ip, YT (2007) Toll and IMD pathways synergistically activate an innate immune response in *Drosophila melanogaster.* Mol Cell Biol 27: 4578-88.

52. Paskewitz, S M, Andreev, O, and Shi, L (2006) Gene silencing of serine proteases affects melanization of Sephadex beads in *Anopheles gambiae.* Ins Biochem Mol Biol 36: 701-11.

53. Volz, J, Osta, MA, Kafatos, FC and Muller, HM (2005) The roles of two clip domain serine proteases in innate immune responses of the malaria vector *Anopheles gambiae.* J Biol Chem 280: 40161-8.

54. Blandin, S and Levashina, E (2004) Thioester-containing proteins and insect immunity. Mol Immunol 40: 903-8.

55. Shiao, SH, Whitten, MM, Zachary, D, Hoffmann, JA and Levashina, EA (2006) Fz2 and cdc42 mediate melanization and actin polymerization but are dispensible for Plasmodium killing in the mosquito midgut. PLoS Pathog 2: e133.
