# Supplementary material for: Caspar Controls Resistance to Plasmodium falciparum in Diverse Anopheline Species
Source: PLoS Pathog. 2009 Mar 13;5(3):e1000335. doi: 10.1371/journal.ppat.1000335 (PMC2647737; doi:10.1371/journal.ppat.1000335)
Supplement: Table S1 — Gene expression in cactus-, caspar-, cactus/rel1- and caspar/rel2-silenced mosquitoes. (0.03 MB PDF) [file ppat.1000335.s003.pdf]

Table S1: Gene expression in *cactus*-, *caspar*-, *cactus/rel1*- and *caspar/rel2*-silenced mosquitoes

A. Comparisons of array-derived and real time PCR-derived gene expression values in *cactus*-silenced mosquitoes and real time PCR-derived gene expression values in *cactus/rel1* double silenced mosquitoes.

**Gene**	*Array ratio*	*Single KD ratio*	*Double KD ratio*
Tep1	2.66	2.51	**0.70**
Cec1	2.75	2.23	**0.60**
CLIPA9	2.75	1.85	0.92
Tep3	3.01	3.25	1.43
Def1	2.99	4.23	0.54
ClipD3	0.60	0.14	0.23
CathD	2.79	3.14	0.28
7024	0.24	0.37	3.06
Fz2	2.73	2.43	1.12
2325	0.67	0.89	0.37
7709	0.27	0.09	0.59
LRRD7	2.66	2.87	0.02
Gam	1.39	1.71	0.03
Fbn37	2.62	6.28	0.29
5159	0.67	1.27	0.05
Fbn9	1.01	1.01	2.04
cec3	3.01	1.91	0.84
Irim1	0.96	1.18	
202	0.65	1.04	1.27

B. Comparisons of array-derived and real time PCR-derived gene expression values in *caspar*-silenced mosquitoes and real time PCR-derived gene expression values in *caspar/rel2* double silenced mosquitoes.

**Gene**	*Array ratio*	*Single KD ratio*	*Double KD ratio*
Tep1	1.42	2.02	**1.63**
Cec1	2.28	2.50	**0.90**
CLIPA9	2.13	2.27	0.39

Tep3	1.74	4.82	1.98
Def1	2.25	3.03	0.45
ClipD3	0.65	0.87	0.07
CathD	0.65	0.85	0.13
7024	0.80	0.55	0.39
Fz2	1.85	3.56	0.49
2325	0.47	0.75	0.94
7709	1.24	1.07	2.82
LRRD7	0.50	0.40	0.69
Gam	1.26	2.00	0.38
Fbn37	1.92	3.43	0.31
5159	1.05	1.01	1.20
Fbn9	0.50	0.23	3.15
Cec3	2.89	2.17	0.35
Irim1	1.19	1.07	
202	0.54	0.59	1.03
PGRP-LC	0.87	0.97	
