# Supplementary material for: Caspar Controls Resistance to Plasmodium falciparum in Diverse Anopheline Species
Source: PLoS Pathog. 2009 Mar 13;5(3):e1000335. doi: 10.1371/journal.ppat.1000335 (PMC2647737; doi:10.1371/journal.ppat.1000335)
Supplement: Table S2 — Immune genes regulated by negative regulator silencing. (0.03 MB PDF) [file ppat.1000335.s004.pdf]

Table S2: Immune genes regulated by negative regulator silencing

A: Immune genes regulated by *cactus* silencing (microarray data)

Gene	Transcript ID	Gene Mean (log transformed)
TEP15	ENSANGT00000019522	-1.19499
PPO1	ENSANGT00000016955	-0.87914
CLIPD3	ENSANGT00000015747	-0.73088
SCRASP1	ENSANGT00000021796	0.913118
LYSC7	ENSANGT00000018439	0.975108
IKK2	ENSANGT00000014138	1.044782
cysteine protease	ENSANGT00000013730	1.093077
SRPN5	ENSANGT00000010507	1.178443
SCRB5	ENSANGT00000010275	1.19922
Peroxidase	ENSANGT00000010957	1.227552
Serine protease	ENSANGT00000027174	1.243046
Hemolymph glycoprotein precursor 2	ENSANGT00000021546	1.262992
Bax Inhibitor-like	ENSANGT00000018745	1.336844
Serine protease	ENSANGT00000013945	1.371173
Thioredoxin peroxidase	ENSANGT00000019782	1.395342
CLIPA9	ENSANGT00000012706	1.415413
Cecropin 1	ENSANGT00000011957	1.455392
CathepsinD	ENSANGT00000013568	1.475551
Programmed Cell Death 4	ENSANGT00000022035	1.48032
Serine protease	ENSANGT00000013929	1.486222
Defensin 1	ENSANGT00000015621	1.583707
Cecropin 3	ENSANGT00000011995	1.588534
TEP3	ENSANGT00000016283	1.59017
CLIPB17	ENSANGT00000022148	1.626139
CLIPD4	ENSANGT00000016188	1.750059
DSCAM10.27	DSCAM exon 10.27	-1.05664
FBN	ENSANGT00000011478	-1.10248

FBN	ENSANGT00000026109	-0.75895
FBN	ENSANGT00000022610	1.13194
FBN37	ENSANGT00000014402	1.390773
LRR	ENSANGT00000028101	-1.15935
LRRD7 (aka APL2)	ENSANGT00000021822	1.410297

B. Immune genes regulated by *caspar* silencing (microarray data)

**Gene**	**Transcript ID**	**Gene Mean (log transformed)**
Serine protease	ENSANGT00000010335	-0.97068
T028113	ENSANGT00000028113	-0.89195
TEP3	ENSANGT00000016283	0.80036
CLIPB4	ENSANGT00000023726	0.831363
CLIPD4	ENSANGT00000016188	0.87801
SCRB1	ENSANGT00000015893	0.92863
CLIPA9	ENSANGT00000012706	1.08824
AgMDL8	ENSANGT00000016928	1.136152
Defensin 1	ENSANGT00000015621	1.170995
Cecropin 1	ENSANGT00000011957	1.188302
Cecropin 3	ENSANGT00000011995	1.52728
Serine protease	ENSANGT00000013929	1.604603
DSCAM10.21	DSCAM exon 10.21	0.948289
FBN9	ENSANGT00000011248	-0.99312
FBN37	ENSANGT00000014402	0.943292
FBN	ENSANGT00000022867	0.972901
LRRD7 (aka APL2)	ENSANGT00000021822	-0.99117
